# The Advancing Roles of Exosomes in Breast Cancer

**DOI:** 10.3389/fcell.2021.731062

**Published:** 2021-11-01

**Authors:** Xi Wang, Chunxiao Sun, Xiang Huang, Jun Li, Ziyi Fu, Wei Li, Yongmei Yin

**Affiliations:** ^1^Department of Oncology, The First Affiliated Hospital of Nanjing Medical University, Nanjing, China; ^2^The First School of Clinical Medicine, Nanjing Medical University, Nanjing, China; ^3^Nanjing Maternity and Child Medical Institute, Obstetrics and Gynecology Hospital, Nanjing Medical University, Nanjing, China

**Keywords:** exosomes, breast cancer, tumor microenvironment, resistance, clinical applications

## Abstract

Breast cancer (BC) develops from breast tissue and is the most common aggressive malignant tumor in women worldwide. Although advanced treatment strategies have been applied and reduced current mortality rates, BC control remains unsatisfactory. It is essential to elucidate the underlying molecular mechanisms to assist clinical options. Exosomes are a type of extracellular vesicles and mediate cellular communications by delivering various biomolecules (oncogenes, oncomiRs, proteins, and even pharmacological compounds). These bioactive molecules can be transferred to change the transcriptome of target cells and influence tumor-related signaling pathways. Extensive studies have implicated exosomes in BC biology, including therapeutic resistance and the surrounding microenvironment. This review focuses on discussing the functions of exosomes in tumor treatment resistance, invasion and metastasis of BC. Moreover, we will also summarize multiple interactions between exosomes and the BC tumor microenvironment. Finally, we propose promising clinical applications of exosomes in BC.

## Introduction

### Breast Cancer

According to Globocan estimates, breast cancer (BC) is one of the most commonly diagnosed cancers in both sexes combined and is the leading cause of cancer death among females. New data published in 2020 suggest that 30% of women in United States will develop BC during their lifetime, and 15% of patients will die from it (Siegel et al., 2020). In addition, it has a morbidity rate of 27.8% and a 15% mortality rate in women worldwide according to a global cancer estimate (Bray et al., 2018).

Breast cancer is a heterogeneous disease that exhibits extensive genomic, transcriptomic, and proteomic dysregulation. With diverse gene expression of steroid hormone receptors [estrogen receptors (ER), progesterone receptor (PR)] and human epidermal growth factor receptor 2 (HER2), and BC is classified into four molecular subtypes. Such heterogeneity has resulted in marked differences in therapeutic options (Harbeck and Gnant, 2017; Table 1).

**TABLE 1:** Different classifications of BC.

Subtypes	Classification	HER2	Hormone receptor
HR positive and HER2 negative	Luminal A-like	HER2 negative	ER or PR positive, or both, low proliferation (low Ki67)
	Luminal B-like	HER2 negative	ER or PR positive, or both, high proliferation (high Ki67)
HER2-enriched	Non-luminal	HER2 positive	ER and PR negative
	Luminal	HER2 positive	ER or PR positive, or both
Triple-negative	TNBC	HER2 negative	ER and PR negative

### Exosomes

Exosomes are a separate type of endosome-released nanometer-sized extracellular vesicles released by all kinds of eukaryotic cells. Vesicles originating from various cells are classified into two main types, exosomes and ectosomes, all of which are cell-derived vesicles that are enclosed by lipid bilayers, with diameters ranging from 30 to 2,000 nm (Kalluri and LeBleu, 2020). The process of exosome generation and secretion is very complicated. Here, we briefly introduce and compare differences in this sophisticated procedure between exosomes and ectosomes. Exosomes are biopolymers that range from 30 to 100 nm in diameter, have unique structural and mechanical characteristics and are formed by two invaginations of the plasma membrane. The first invagination occurs along biomolecules associated with the extracellular milieu and forms an intracellular cup-shaped structure called the early-sorting endosomes, which subsequently mature into late-sorting endosomes. Intracellular multivesicular bodies containing intraluminal vesicles are formed in the subsequent invagination after receiving late-sorting endosomes. From early to terminal endosome maturation, multivesicular bodies are processed either by fusing with the plasma membrane to undergo exocytosis of the contained intraluminal vesicles (namely, exosomes) into the extracellular space or fusing with lysosomes or autophagosomes to be degraded (Kalluri and LeBleu, 2020). In contrast to exosomes, ectosomes, including microvesicles, microparticles, and large vesicles, bud directly and pinch off from the plasma membrane and are extremely heterogeneous in size (Cocucci and Meldolesi, 2015).

Exosomes are distributed through human body fluids to transport their cargos to cells in the vicinity or dwelling remotely, which brings notable advantages to detecting potential pathological conditions. The uptake mechanisms of exosomes are widely discussed, including direct plasma membrane fusion and endocytosis, such as micropinocytosis (Nakase et al., 2015) and phagocytosis (Feng et al., 2010).

Exosomes are secreted by almost every kind of cell and play an important role in cell-cell communication (van Niel et al., 2018). They contain and deliver numerous biomolecules, ranging from multiple proteins, lipids, DNA, microRNAs (miRNAs), circular RNAs (circRNAs), messenger RNAs (mRNAs), long non-coding RNAs (lncRNAs), and metabolites, to target cells. Intriguingly, exosome contents may vary, as distinct proteins and nucleic acids are enclosed according to their cell of origin (Wen et al., 2019). However, the selection mechanisms for content loading remain unveiled. In terms of cancers, an increasing number of articles have reported exosome involvement in cancer development and function and as potential vectors to aid in improving therapeutic strategies. Exosomes may also have profound impacts on the tumor microenvironment, as they represent intensively specialized entities of interaction carrying specific surface markers, oncogenic proteins and other biomolecules that can be transferred horizontally into stromal cells. Consequently, the tumor microenvironment is altered to allow for tumor progression (Rak and Guha, 2012). As it is difficult to assign the correct nomenclature to lipid bilayer particles secreted by cells in diverse experimental conditions, according to the claim of the “International Society for Extracellular Vesicles” (Théry et al., 2018), some scientific reports referred to “extracellular vesicles” as an unified term covering small extracellular vesicles containing exosomes. Thus, in this present review, several findings related to extracellular vesicles in the discussion of metastatic BC were also presented.

### Tumor Microenvironment

The tumor microenvironment often refers to a dynamic region intrinsic to the solid tumor. In addition to tumor cells, it also embodies multiple cell types and non-cancerous components such as fibroblasts, immune-related cells (e.g., tumor associated macrophages, dendritic cells, mesenchymal stem cells, and T cells), adipocytes and endothelial cells. These cells each with diverse biological contributions constitute an integral part of the unique microenvironment for BC cells. Meanwhile, they have crucial roles in local interact with BC progression. For example, cancer-associated fibroblasts are believed to suppress BC microenvironment immunity and was exemplified in the work undertaken by Costa et al. (2018). One of the four cancer-associated fibroblasts subsets which called CAF-S1, inhibited T effector proliferation by enhancing the regulatory T cell capacity.

Also, the tumor microenvironment provides a milieu for communication between cancer cells and surrounding immune cells, fibroblasts, endothelial cells, and adipocytes. Interactions between malignant cells and non-malignant cells in the tumor microenvironment occur via exosomes, soluble cytokines or signaling molecules that cancer cells are capable of manipulating to their own advantage in processes such as angiogenesis, immune evasion, migration, metastasis, and drug resistance (Meads et al., 2009). Under the modulation of the tumor microenvironment, non-tumor cells can be affected by tumor-related active substances, forcing them to constantly adapt, and creating an environment that facilitates tumor growth (Quail and Joyce, 2013). For example, normal fibroblasts can be stimulated to become cancer-associated fibroblasts and create a specific extracellular matrix to support tumor cell migration (Chen et al., 2017a). Diverse immune and inflammatory molecules in the tumor microenvironment also block the immune killing response, empowering tumor cell survival (Toor et al., 2019). In addition, the hypoxic tumor microenvironment that is generated and its primary effectors represent a critical step in the tumorigenic process (Petrova et al., 2018). Together, these cancer-associated activities in the tumor microenvironment play detrimental roles in favoring neoplasm malignant development (Jarosz-Biej et al., 2019).

In this review, we summarize the multifaceted functions of exosomes in BC therapeutic resistance, invasion, and mediation of the tumor microenvironment to better understand and treat related malignant progression. In addition, their potential applications in diagnosing and providing novel clinical therapeutic options are also discussed in the section “Conclusion.”

## The Effects of Exosomes in the Breast Cancer Tumor Microenvironment

The tumor microenvironment is often described as the soil where tumor cells grow, which is formed by a variety of normal cells, signal-transmitting molecules, blood vessels, and extracellular matrix (Quail and Joyce, 2013). Consequently, cancer and other cells dwelling in the same niche are involved in multiple modes of interactions to synchronize and antagonize the negative influence of environmental changes, such as drug resistance (Diaz Bessone et al., 2019). In detail, surrounding normal cells, including immune-associating cells, vascular endothelial cells, adipocytes, fibroblasts, and bone-marrow mesenchymal stromal cells, are recruited to and have prominent roles in cancer (Kalluri and Zeisberg, 2006). For instance, epithelial-mesenchymal transition is necessary during embryonic development. However, findings found that various alterations in glycolysis, oxidative phosphorylation, and autophagy in this process promoted tumor multidrug resistance (Erin et al., 2020). Many tumor cells survive by developing immune evasion mechanisms, including secretion of neoplastic proteins, escape from T-cell attack, promotion of T regulatory cells, and decreased levels of antigen-presenting proteins. Studies have revealed that BC cell-released exosomes are capable of affecting the immune system through interplay with T-cells, regulatory T-cells, dendritic cells, and macrophages.

### The Effects of Exosomes on Immune System Regulation

**FIGURE 1:**
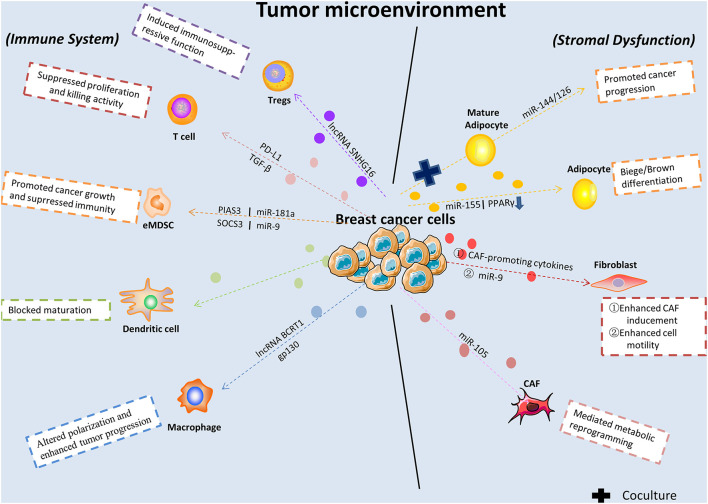
Breast cancer-released exosomes in regulating immune system, fibroblasts, and adipocytes in tumor microenvironment. BC-derived exosomes are released to alter immune system and cause stromal cell dysfunction in tumor microenvironment via transferring multifarious cargos and targeting genes or signaling pathways. eMDSC, early-stage myeloid-derived suppressor cells; CAF, cancer associated-fibroblast; Tregs, regulatory T cells.

Immunoediting in cancer is a dynamic process, which further determines tumor progression. The distinct modes between immune cells and cancer cells result in widely different outcomes. Immune cells like dendritic cells play a central role in immune responses against cancer. On the contrary, infiltration by plasmacytoid dendritic cells was found correlates with decreased overall survival and poor relapse-free survival in BC (Treilleux et al., 2004). Besides, macrophages-2 released CHI3L1 was found prompted tumor progression, which revealed a novel field of macrophages with respect to BC metastasis (Chen et al., 2017b). In this part, we will focus on describing the effects of exosomes on BC immune system regulation. Tumor-derived exosomes contain molecules that can stimulate immune cell dysfunction and transform the surrounding microenvironment into a suitable niche conducive to their growth and metastasis (Figure 1). Recently, one study revealed that lncRNAs in exosomes secreted from BC were correlated with macrophage polarization. Specifically, lncRNA BCRT1 mediated by exosomes promoted M2 polarization of macrophages, which further accelerated BC progression (Liang et al., 2020). Similarly, a previous work also demonstrated that macrophage polarization in the cancer-related microenvironment was altered by BC-derived exosomes, partially through the glycoprotein 130-STAT3 signaling pathway (Ham et al., 2018). Furthermore, the progression of early-stage myeloid-derived suppressor cells was promoted by tumor exosome-derived miR-9 and miR-181a by activating the JAK/STAT signaling pathway in BC (Jiang et al., 2020).

**FIGURE 2:**
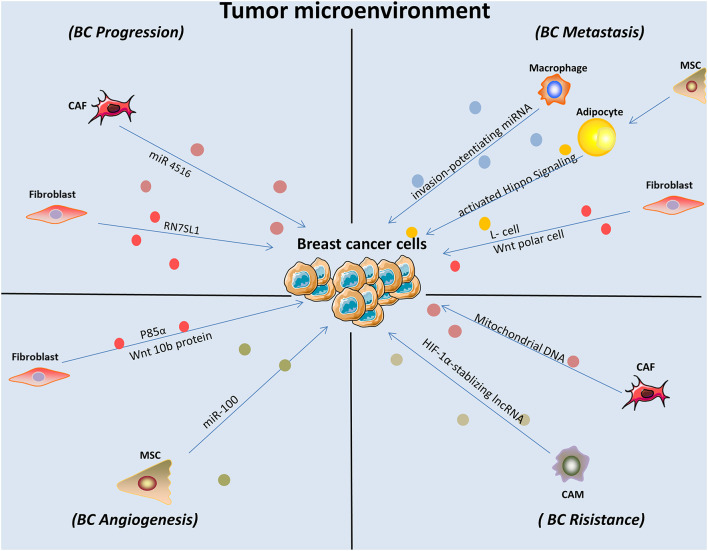
Exosomes derived from stromal cells affected BC cell behaviors and contributed to enhanced malignant progression. CAM, cancer associated macrophage; MSC, mesenchymal stem cells; CAF, cancer associated fibroblast.

Meanwhile, the function of exosomes that arise from the tumor microenvironment on the malignant progression of BC cells is also significant (Figure 2). It was demonstrated that cancer-related macrophages in the tumor microenvironment contributed to enhanced BC cell aerobic glycolysis and apoptotic resistance by transmitting extracellular vesicles encompassing a distinctive lncRNA. This HIF-1α-stabilizing lncRNA was clinically positively associated with poor chemotherapeutic responses and shorter survival in BC patients (Chen et al., 2019). In addition, BC cells can reside in the bone marrow and remain dormant for decades. However, how macrophages modulate dormancy is still poorly understood. One study conducted a survey and found that exosomes released by macrophages exhibiting an M1 phenotype activated NF-κB to reverse silent BC cells into cycling cells, which contributed to BC progression (Walker et al., 2019). Another study concluded that macrophages played an important role in the regulation of BC metastasis by transmitting exosome-mediated invasion-potentiating miRNA (Yang et al., 2011).

Immunological surveillance plays a vital role in the generation and maintenance of the anti-tumor function of the immune system (Schreiber et al., 2011), and studies have sought to investigate the role of exosomes in its development and subsequent outcomes. For instance, Yang et al. (2018) demonstrated that BC-released exosomes were capable of blunting T-cell activation and ablating activities by expressing programmed cell death-1 on their surface, effectively helping tumor cells evade immune surveillance. After bypassing the immune system, tumor cells are potentiated to successfully seed and colonize various distant organs, such as the lung. Exosomes secreted by highly metastatic murine BC cells inhibit anti-tumor immune responses in premetastatic organs, directly suppressing T-cell proliferation and suppressing NK cell cytotoxicity. Hence, this process contributes to promoting metastasis (Wen et al., 2016). A recent finding reported that a unique lncRNA, SNHG16, enclosed by exosomes was secreted from BC cells and induced CD73 + γδT1 cells to act as immunosuppressive regulatory T-cells by activating the TGF-β1/SMAD5 pathway (Ni et al., 2020).

As discussed above, the BC tumor microenvironment contains specific immune sub-populations such as immune surveillance evasion and immune regulation contributed to inhibited anti-tumor immune response of host cells. Several anti-inflammatory mechanisms presented by reciprocal interactions between BC cells and tumor microenvironment by utilizing exosomes as mediators have been summarized. In section “Conclusion,” the above findings demonstrated that blocking cancer-derived exosomes may have favorable impacts for treating BC.

### The Effects of Exosomes on Fibroblast Cells

Fibroblasts are known as non-vascular, non-inflammatory, and non-epithelial cells in surrounding tissue. Their secretion of growth factors, various chemokines and extracellular matrix facilitates recruiting the endothelial cells and pericytes. They are also found activated into cancer-associated fibroblasts and become a key determinant in the malignant progression of tumor including metastasis, invasion, resistance, and imply an important target in cancer treatments (Plava et al., 2019). As pioneering studies suggested that tumor cells can be promoted in an wound-repair-associated microenvironment. It is vital for us to deepen our understanding in the field of reciprocal effects between fibroblast and tumor microenvironment (Bissell and Hines, 2011). A recent finding revealed the PERK signaling was selectively activated by Rho-associated kinase which caused fibroblast reprogramming and contributed to BC progression (Boyle et al., 2020). Furthermore, fibroblast-expressed PIK3Cδ was found promoting TNBC progression with the help of a high-throughput siRNA kinome screening technology and recognized as a potential target (Gagliano et al., 2020). The tumor microenvironment forms the core site in which neoplastic cells, stromal fibroblasts and cancer-associated fibroblasts interact with each other, and exosomes play a complex role in that interplay (Figure 2). For example, endogenous RNAs must remain shielded under non-pathological conditions. However, one study showed that RN7SL1, an endogenous RNA normally covered by RNA binding proteins SRP9/14, was increased in stromal fibroblast exosomes in response to triggering stromal NOTCH-MYC. After being transferred to ISG-R BC cells in exosomes and then bound to RIG-I, this unshielded RN7SL1 exosomal RNA promoted aggressive features of cancer (Nabet et al., 2017). Additionally, a study demonstrated that stromal fibroblasts are converted into cancer-associated fibroblasts with low expression of p85α. Furthermore, p85α-deficient fibroblast-derived exosomes carrying the Wnt10b protein were transferred into BC cells, which induced epithelial-to-mesenchymal transition and was consequently involved in breast malignant progression (Chen et al., 2017a). Another study established that the horizontal transfer of exosomal mitochondrial DNA derived from cancer-associated fibroblasts exerted its function as a tumorigenesis signal in hormonal therapy-resistant disease. The specific mechanisms underlying the transfer include promoting escape from metabolic quiescence of therapy-mediated cancer stem-like cells and further contributing to hormonal therapy resistance in oxidative phosphorylation-dependent BC cells (Sansone et al., 2017b). In addition, a specific exosomal miRNA-4516 was recently found to be involved in intercellular crosstalk. The miRNA secreted from cancer-associated fibroblasts was correlated with the regulation of triple-negative BC (TNBC) progression. Specifically, reduced expression of miRNA-4516 promoted proliferation and malignancy in TNBC by targeting FOSL1, which exerted its function as a tumor progression mediator and thus contributed to tumor development (Kim et al., 2020). A recent study that centered on the relationship between cancer-associated fibroblasts and BC cell activities was conducted in a BC mouse model and demonstrated that after ablation of focal adhesion kinase, expression of exosomal miRNA-16, and 148a was increased in cancer-associated fibroblasts, which inhibited migration and metastasis in BC (Wu et al., 2020). A previous study established that exosomes derived from a fibroblast cell line, L cells, stimulated BC cell lines (MDA-MB-231 and SUM-159PT) to exhibit protrusive activity, motility, and lung metastasis through the Wnt-planar cell polarity pathway. Specifically, it was revealed by the authors in subsequent studies that fibroblast-released exosomes co-localized with BC cell-derived Wnt11 within endocytic vesicles after uptake in target cells, which was vital for enhancing exosome-stimulated motility in BC cell lines (Luga et al., 2012). This direct evidence demonstrated that exosomes play a part in mediating metastatic properties between stromal and BC cells.

Another investigation found that exosomal miRNA-9 was transmitted from BC cells to normal fibroblasts and resulted in enhanced cell motility. Furthermore, this miRNA was also released by fibroblasts and was in turn capable of altering tumor cell behavior via extracellular matrix remodeling pathways (Baroni et al., 2016). Additionally, extracellular vesicles derived from BC containing miRNA-105 activated oncoprotein MYC signal transduction. This result triggered metabolic reprogramming of cancer-associated fibroblasts by conditioning their metabolic features in different metabolic environments and sustained tumor growth (Yan et al., 2018; Figure 1).

The underlying mechanism of how normal fibroblasts were reached and transformed by cancer-associated fibroblasts-derived promoting cytokines is unclear. Closer investigation into this question found that promoting cytokines gained the ability to effectively break the extracellular matrix barrier after loading into exosomes and reached normal fibroblasts during the early stages of BC development. Diffusion of exosomes was enhanced with the aid of extracellular matrix fibril alignment in a force-dependent manner (Jung et al., 2020).

### The Effects of Exosomes on Angiogenesis

Mesenchymal stem cells was first described as multipotent hematopoietic bone marrow cells to give rise to bone, marrow stroma, adipocytes, muscle, and connective tissue (Caplan, 1991). Reduction of tumor growth by mesenchymal stem cells can be modulated by inhibiting angiogenesis, P2X signaling was implied as a pathway promoting mesenchymal stem cells mediated BC cell proliferation (Maffey et al., 2017). Endothelial cells are also important players in the angiogenic promotion of cancer growth and metastatic spreading. A study found that the exchange of growth factors between co-cultured endothelial and BC cells are capable of modulating the angiogenic response and tumor invasion ability directly (Zhang et al., 2018). The shuttling of miRNA-100 from human mesenchymal stem cells to BC cells via exosomes, which suppressed angiogenesis in recipient cells, was substantiated by a previous study. This process was induced by modulating the mTOR/HIF-1α/VEGF signaling axis in BC cells (Pakravan et al., 2017). Annexin II was identified as one of the most highly encapsulated proteins in exosomes using proteomics profiling and was found to be significant in BC pathogenesis. One study demonstrated that exosomal Annexin II promoted tPA-dependent angiogenesis both *in vitro* and *in vivo*, probably by functioning as a specific receptor for both tPA and plasminogen (Maji et al., 2017). Furthermore, the level of exosomal Annexin A2 was found elevated in the serum of African-American TNBC BC women, which also correlated with intensive possibility of promoting angiogenesis (Chaudhary et al., 2020). Mesenchymal stem cell-derived exosomes contain protein and RNA profiles that are different from their donor cells and can be internalized by BC cells. Upon delineating the mechanism, mesenchymal stem cell-derived exosomes contained a specific miRNA-16, which targeted vascular endothelial growth factor and drastically downregulated its expression. As a result, the inhibition of angiogenesis in BC was induced both *in vitro* and *in vivo* (Lee et al., 2013). After treatment with docosahexaenoic acid, expression of exosomal miRNAs was increased in MCF7 BC cells. Mechanistically, with the application of drug-treated BC-derived exosomal miRNAs to endothelial cell cultures, researchers found that tube formation was significantly decreased, suggesting that BC exosome signaling was targeted by docosahexaenoic acid to inhibit angiogenesis (Hannafon et al., 2015). In addition, a study focusing on the role of exosomes isolated from TNBC cells in cancer progression compared differences between exosomes secreted from a less invasive cell line. It was found that exosomes derived from more invasive TNBC cells promoted endothelial tubule formation after uptake, which positively correlated with vasculogenesis and subsequent angiogenesis in secondary cells (O’Brien et al., 2013).

### The Effects of Exosomes in the Interactions Between Adipocytes and Breast Cancer Cells

It was demonstrated that the co-cultivation of BC cells with adipocytes contributed to elevated invading capacity both *in vitro* and *in vivo* (Wu et al., 2019a). According to recently reported review, abundant factors are participated in the pro-tumorigenic functions of adipocytes, including production of adipokines and other soluble factors (Choi et al., 2018). Also, obesity and adipocytes was reported strongly connected with poor prognosis of patients with BC (Picon-Ruiz et al., 2017). In this section we will give a brief summarize of the pivotal roles of adipocytes in BC cell growth and progression (Figure 2). A previous study demonstrated that exosomes derived from pre-adipocytes played important roles in regulating early-stage BC cell activities. After treatment with the natural anti-tumor compound shikonin, miRNA140 expression in exosomes increased and targeted SOX9 signaling, which may consequently block differentiation, stemness, and migration in the BC microenvironment (Gernapudi et al., 2015). It was also noted that adipocytes derived from mesenchymal stromal/stem cell-released exosomes promoted proliferation and migration after incorporation into the BC cell line MCF7 through the Hippo signaling pathway. Furthermore, these exosomes protected MCF7 cells from serum derivation or chemotherapy-induced apoptosis *in vitro* (Wang S. et al., 2019). A novel study investigated exosomes derived from cocultivation of BC cells with mature adipocytes and their effects on tumor progression. Mechanistically, researchers demonstrated that exosomal miRNA-144 and miRNA-126 were found induced beige/brown differentiation and reprogrammed metabolism in recipient adipocytes surrounding the tumor cells to facilitate BC progression (Wu et al., 2019b). In addition, cancer-related cachexia is a metabolic complication of cancer, and miRNA in exosomes secreted from BC cells plays a fundamental role in this process and consequently promotes cancer progression. BC cell-derived exosomes transferred miRNA-155 into adipocytes, where it exerted its function by promoting beige/brown differentiation and remodeling metabolism in resident adipocytes by downregulating PPARγ expression (Wu et al., 2018; Figure 1).

### Other Extracellular Vesicles and Cytokines in Regulating Tumor Microenvironment

As exosomes are originated from extracellular vesicles which also play crucial roles in intercellular communications, in this paragraph we will focus on describing other extracellular vesicles and cytokines such as microvesicles, apoptotic bodies and their potential roles in regulating malignant process especially in BC tumor microenvironment. An investigation illuminated stromal microvesicles mediated evolution of cancer stem-like cells by transferring genetic material from local stromal cells to cancer cells. As a result, this horizontal transfer triggered the development of therapy-resistant metastases in BC (Sansone et al., 2017a). Furthermore, a recent study revealed that *O*^2^-3-Aminopropyl diazeniumdiolates 3a–f was created to markedly suppress the metastatic ability of TNBC by attenuating microvesicles formation in an NO-dependent manner (Kang et al., 2018). Welte et al. (2016) highlighted that the mTOR pathway promoted G-Colony-stimulating factors expression in preclinical models of BC which further resulted in immunosuppressive tumor microenvironment.

## Roles of Exosomes in BC Drug Resistance and Radioresistance

The resistance of BC cells to chemotherapy, endocrine therapy, anti-HER-2-targeted therapy, and radiotherapy currently limits their treatment efficacy. As a result, scrutiny of the underlying mechanisms leading to resistance is essential to develop more effective therapies for patients with BC. As exosomes are involved in intercellular communication by transmitting their cargos not only to recipient cells in their immediate surroundings but also to distant organs, it is reasonable to envisage their role in inducing or improving the resistance of BC cells. The contents of exosomes are highly diverse, in which several kinds of RNAs, proteins, DNAs have been detected, including oncoproteins, DNA fragments, mRNAs and non-coding RNAs, such as miRNAs, tRNA, snRNA, long-non-coding RNA (lncRNA), and circular RNA (circRNAs) (Murillo et al., 2019). Mastering our understanding of the underlying resistance mechanisms will aid in enhancing cancer treatments and subsequently improving patient outcomes. The explicit mechanisms by which resistance may exist are outlined in this section.

### Drug Resistance

Recent evidence has revealed that exosomes play a major role not only in regulating drug resistance but also in transferring resistance to drug-sensitive BC cells. Furthermore, resistance can be inherent in the tumor cell itself or initiated by treatment with anticancer drugs (acquired resistance) and results in poor prognosis in cancer patients (Pereira et al., 2020). Clear and definite individual causes underlying chemoresistance could facilitate the development of more appropriate therapies for each patient. In this section, we discuss how transmission of exosomes induces the development of BC drug resistance and how molecular insights into exosome contents have unraveled their pivotal roles.

#### Exosomal Non-coding RNAs in Breast Cancer Drug Resistance

A growing volume of literature during the past decade indicates that non-coding RNAs are not the previously commonly deemed non-functional “junk” (Slack, 2006). Indeed, these RNAs, despite not being involved in encoding proteins, are mysterious regulators of most and appear to exert crucial functions in a wide range of human diseases, notably cancer. They have been identified as both oncogenic drivers and tumor suppressors that influence cancer progression in complex ways (Slack and Chinnaiyan, 2019). Non-coding RNAs are divided broadly by their size. Small non-coding RNAs, such as miRNAs, are capable of regulating mRNAs through binding to complementary sequences, while lncRNAs, including subtypes such as pseudogenes and circRNAs, are essential for creating a flexible molecular scaffold to sustain cellular activities (Anastasiadou et al., 2018). On the meantime, studies focused on investigating the effects of tRNA and its fragment deepened our understanding of BC malignant process. A tRNA fragment 5′-tiRNA^Val^ was demonstrated capable of suppressing BC development through inhibiting FZD3/Wnt/β-Catenin signaling pathway, which could serve as a potential diagnostic biomarker (Mo et al., 2019). Although, there do not have adequate evidence demonstrate the relation between tRNA and BC in the aspect of exosomes, advancements of high-throughput sequencing technology may aid in better identifications of their roles and mechnisms in cancer.

Exosome cargo includes several types of nucleic acids, including miRNAs, lncRNAs and mRNAs. Substantial evidence indicates that exosomes are crucially involved in the development of BC drug resistance by transmitting RNA. For instance, one finding indicated the possibility that drug-resistant BC cells conferred resistance to drug-sensitive BC cells partly by releasing specific exosomal miRNAs and thereby enhancing the general resistance of MCF-7/S after co-culture (Chen et al., 2014). Specifically, the drug resistance trait was induced after transfer of exosomal miR-100, miR-222 and miR-30a from resistant BC cell lines MCF-7/Adr and MCF-7/Doc to MCF-7/S recipient sensitive cells. However, the underlying mechanisms of these effects have yet to be elucidated. Wei et al. (2014) demonstrated that specific exosomal miRNA-221/222 secreted by tamoxifen-resistant ER-α-positive BC cells reduce expression of P27 and ER-α in tamoxifen-sensitive BC cells, resulting in the transfer of drug resistance (Wei et al., 2014). Furthermore, researchers found that extracellular vesicles originating from HCC1806 TNBC cells have the ability to induce proliferation and transfer drug resistance to non-tumorigenic MCF10A breast cells, which is potentially mediated by alterations in gene and miRNA expression correlated with cell proliferation, invasion, and migration (Ozawa et al., 2018). Similarly, a recent study focused on exploring Adriamycin resistance in BC cells, revealing that exosomal miR-221-3p was the key in promoting resistance by targeting PIK3R1 (Pan et al., 2020). In addition, exosomes may modulate resistance and migration capacity from chemoresistant BC cells to sensitive cells, partly by the encapsulation and transfer of miR-155, which is also associated with cancer stem cells and the process of epithelial-to-mesenchymal transition (Santos et al., 2018). Notably, according to recent studies, various factors or pathways are amended to impair the efficiency of chemotherapeutic agents and induce drug resistance. For example, stimulation of the cancer stem-like cell phenotype is closely related to chemoresistance in tumors by differentiation into different cell types (Das and Law, 2018). Chemotherapy induces BC cells to secrete multiple extracellular vesicle encapsulated miRNAs, including miRNA-9-5p, miRNA-195-5p, and miRNA-203a-3p, which led to adaptation of cancer stem-like cell traits by simultaneously targeting the transcription factor One Cut Homeobox 2. In turn, downregulation of these miRNAs or upregulation of One Cut Homeobox 2 expression abolished the cancer stem-like cell-activating effect of extracellular vesicles from chemotherapy-treated BC cells (Shen et al., 2019).

While BC cell malignant activities may be modulated by lncRNAs, the underlying drug resistance mechanisms within exosomes are still largely unknown. A recent study indicated that the lncRNA AFAP1-AS1 is highly expressed in trastuzumab-resistant BC cells. Notably, this specific lncRNA disseminated trastuzumab resistance to other BC cells through transmission in exosomes to further associate with AUF1 and activated ERBB2 translation, which did not influence mRNA expression (Han et al., 2020a). Another lncRNA with abnormal expression was demonstrated in a recent finding. Doxorubicin has been widely recognized as a first-line drug in treating BC (Mann et al., 2020). LncRNA H19 was found to be strikingly overexpressed in doxorubicin-resistant BC cells and was encapsulated into exosomes to transfer drug resistance into drug-sensitive BC cells. Similarly, downregulation of H19 reversed the chemoresistance of doxorubicin in sensitive BC cells (Wang et al., 2020). The oncogenic function of lncRNA AGAP2-AS1 was previously characterized in gastric cancer (Qi et al., 2017). However, its underlying functions in BC remain unknown. A novel study concluded that AGAP2-AS1 is dysregulated in trastuzumab-resistant BC cells and plays critical roles in enhancing trastuzumab resistance by packaging into exosomes in an hnRNPA2B1-dependent manner and inhibiting trastuzumab-induced cell cytotoxicity (Zheng et al., 2019). In addition, a previous finding verified the association between the lncRNA small nucleolar RNA host gene 14 and the response to trastuzumab in BC cells. Functional experimentation and analysis demonstrated that this lncRNA aided in enhancing trastuzumab resistance possibly by modulating the apoptosis-related signaling pathway and can be exploited as a potential diagnostic biomarker in future development (Dong et al., 2018). Another novel study first compared the exosomal lncRNA urothelial carcinoma-associated 1 loading differences between tamoxifen-resistant cells and tamoxifen-sensitive cells in estrogen-positive BC, revealing significantly increased levels of urothelial carcinoma-associated 1 in tamoxifen-resistant cells, especially in exosomes derived from these cells. These results indicate that the transfer of urothelial carcinoma-associated 1 mediated by exosomes might represent a vital mechanism in inducing acquired tamoxifen resistance in BC cells (Xu et al., 2016).

In addition to the various microRNAs and lncRNAs discussed above, circRNAs have also been detected in exosomes. Although exosomal circRNA and its associated functions have been little studied in BC drug resistance, some articles have revealed exosomal circRNA roles in other types of cancers. For example, the metastatic ability of liver cancer cells was enhanced by transferring exosomal circRNA-100338, affecting human umbilical vein endothelial cells and their proangiogenic activity (Huang et al., 2020). Similarly, hepatocellular carcinoma cell metastatic potential was found to be transferred via exosomes with circPTGR1 and its three isoforms (Wang G. et al., 2019). As the liver is the most common site of metastasis in BC, it is plausible to envisage exosomal circRNAs and their effects on BC progression, and further explorations are warranted in future research.

#### Exosomal Proteins in Breast Cancer Drug Resistance

Various oncogenic proteins and oncometabolites are prominent players in BC progression. Data provided by Mishra et al. (2018) supported that ADHFE1 which is a BC oncogene protein contributed to the accumulation of oncometabolite 2-hydroxyglutarate and induced metabolic reprogramming through MYC signaling. In addition to nucleic acids, protein cargo in exosomes is also involved in the drug resistance of BC. TrpC5, a potential transient receptor protein, prompted the formation of the multidrug efflux transporter P-glycoprotein via a Ca^2 +^ and activated T-cell isoform c3-induced mechanism through accumulation and transfer in extracellular vesicles. In addition, upregulation of exosomal Trpc5 has a unique role in trapping Adriamycin and partially transferring chemoresistance from drug-resistant BC cells to non-resistant cells (Ma et al., 2014). Exosomal delivery of P-gp, a transmembrane protein with a higher concentration in drug-resistant BC cells, caused efflux of drug substrates to maintain sublethal intracellular drug levels. It was suggested to play an important role in transferring drug resistance from docetaxel-resistant cells to drug-sensitive cells in BC (Lv et al., 2014). In addition, a novel study demonstrated that UCH-L1-containing exosomes secreted by Adriamycin-resistant human BC cells were taken up by Adriamycin-sensitive human BC cells in a time-dependent manner and ultimately contributed to the chemoresistance phenotype. This individual trait also entailed potential diagnostic implications for UCH-L1 (Ning et al., 2017). Similarly, Yang et al. (2017) found that glutathione S-transferase P1-containing exosomes have the ability to transfer drug resistance from Adriamycin-resistant BC cells to Adriamycin-sensitive BC cells by the translocation of glutathione S-transferase P1, which is a metabolic enzyme that reportedly decreases the efficiency of several anticancer drugs via conjugation with glutathione (Coles and Kadlubar, 2003). Expression of this transferase was considerably higher in Adriamycin-resistant BC cells and their corresponding exosomes. More importantly, increasing the concentration of this enzyme in circulating exosomes should be explored for clinical prediction applications (Yang et al., 2017).

Unfortunately, chemotherapy stimulates the creation of undesirable products while killing cancer cells. It was demonstrated that intensive chemoresistant therapeutic-induced senescent cells were generated after chemotherapy and helped form stem cell niches that contributed to metastasis. Importantly, increased extracellular vesicles and exosomes containing key proteins involved in tumor progression were harvested from and regulated by therapeutically induced senescent cells in TNBC cells. Additionally, expression of the multidrug resistance protein 1/p-glycoprotein was increased in therapeutically induced senescent cells. Taken together, these finding indicate that extracellular vesicles in therapeutically induced senescent cells TNBC cells are overexpressed and partially aid in conveying resistance to chemotherapy (Kavanagh et al., 2017).

#### Exosomes Involved in Other Mechanisms of Drug Resistance in Breast Cancer

Although various exosomal cargos are closely correlated with BC therapeutic resistance, exosomes themselves are also influenced and play vital roles in impairing BC therapy efficiency. For example, a previous investigation found that exosomes influenced the outcomes of antibody drugs by regulating their binding to tumor cells (Aung et al., 2011). Besides, exosomes released from HER2-overexpressing BC cells containing the HER2 protein neutralized the HER2-targeted antibody-based drug trastuzumab and attenuated its interaction with the intended BC target cells, leading to enhanced aggressiveness (Ciravolo et al., 2012). Intriguingly, exosomes can also function as novel shelters. It was revealed that the epidermal growth factor receptor, which is essential in promoting BC progression, was protected in exosomal compartments, allowing it to evade attacks from epidermal growth factor receptor small inhibitors in a TNBC model. As a result, drug resistance was triggered, and related signaling pathways were stimulated to activate proliferation and migration in TNBC recipient cells (Hung et al., 2019).

### Resistance to Radiotherapy

Radiotherapy also provides an effective option for treating BC; however, resistance to radiotherapy has been identified in large-scale studies. Some findings indicated that exosomes engaged in this process. For example, a study investigating the effect of X-ray irradiation on differences in exosome activity in MCF-7 BC cells found that exosome biogenesis and secretion were activated in a dose-dependent manner, revealing the potential of exosomes to convey resistance to radiotherapy in BC cells (Jabbari et al., 2019). In addition, cargo from irradiated cell-derived exosomes was distinct from that of non-irradiated cells, indicating alterations in the exosomal formation system (Mutschelknaus et al., 2017). However, one study demonstrated that irradiated mouse BC cells secreted exosomes that transferred double-stranded DNA to dendritic cells and stimulated dendritic cell activation of IFN-I, which was vital for recruiting dendritic cells and indicated promising prospects for connecting radiotherapy with immunotherapy in BC. Specifically, exosomes derived from irradiated cells elicited immune responses of tumor-specific CD8 + T-cells and inhibition of tumor size in a mouse BC model (Diamond et al., 2018). These data showed that BC-derived exosomes mediate radioresistance by transferring adverse molecules from irradiated cells toward target cells. However, further investigations are warranted to reveal the underlying roles of BC-derived exosomes in the generation of resistance. Under these circumstances, it is a promising step to develop additional treatment approaches following radiotherapy by interfering with exosomal biogenesis and function.

### Exosomes in Relieving or Reversing Therapy Resistance of Breast Cancer

While many drug-resistance incidents induced by exosomes and their contents in BC cells have been discussed above, it is noteworthy that some findings also revealed that exosomes have a role in relieving or reversing resistance in BC. A novel study identified an agent called d rhamnose β-hederin that attenuates resistance traits in docetaxel-resistant BC cells and reduces tumor burden by decreasing exosome secretion (Chen et al., 2018). In addition, exosomes secreted from multidrug-resistant BC cells were inhibited by psoralen and impaired resistance via the PPAR and p53 signaling pathways. This report also provided novel insight into treating BC resistance to chemotherapy (Wang et al., 2016). In addition, blocking PGE2/EP4 signaling contributed to β-catenin removal from BC stem cells through EVs/exosomes. As a result, the number of chemotherapy-resistant cancer stem cells was reduced, which enhanced chemosensitivity in BC cells (Lin et al., 2018). It was also demonstrated that miRNA-770 can be transferred by exosomes from TNBC cells to tumor-associated macrophages, which sensitize responses to chemotherapy by regulating macrophage polarization (Li Y. et al., 2018). Furthermore, trastuzumab chemoresistance was reversed after transmitting exosomal miRNA-567 into BC cells and suppressing autophagy, which is important in maintaining the survival of cancer cells under various adverse conditions, including nutrient deficiency, chemotherapy, and radiotherapy (Han et al., 2020b).

These data indicated that BC exosomes mediate resistance via multiple different interventions and revealed conceivable values of exosomes as diagnostic and prognostic biomarkers. Nevertheless, clinical trials are needed to further demonstrate the ability to target exosomes and their effects in practical treatment.

## Therapeutic Applications of Exosomes in Breast Cancer

Although developing strategies have been applied to treat BC, including surgical intervention, chemotherapy, radiotherapy, and hormone therapy, challenges have also arisen (Harbeck and Gnant, 2017). Drug resistance, high toxicity and other mechanisms were found to be responsible for treatment failure. To tackle these obstacles, more attention should be paid to identifying novel therapies and delivering technologies for BC. According to this trend, precision oncology has aroused attention of researchers as their potential capability in discovering predicting molecular biomarkers. Currently, exosomes have gained interest as a novel type of cancer vaccine and potential targeted drug delivery vehicle because they significantly inhibit BC progression without obvious toxicity or immunogenicity (Gong et al., 2019). In this section, we discuss the pleiotropic functions of exosomes in BC therapeutics and their novel roles in advanced BC are also described (Table 1).

### Exosomes as Complementary Tools in Liquid Biopsy

In contrast to traditional “solid biopsies,” liquid biopsy provides a non-invasive method to both consistently monitoring and improving our knowledge about the BC metastatic process (Nadal et al., 2012). By analyzing immunosuppressive profiles in liquid biopsy, researchers revealed that elevated level of eMDSCs exacerbated immunosuppression which facilitated identification of poor neoadjuvant chemotherapy BC responders (Salvador-Coloma et al., 2020). Compared with other types of liquid biopsies, exosomes have advantages in different aspects. First, the cup-shaped appearance makes it easier to distinguish exosomes by using electron microscopy in comparison to other subcellular particles such as apoptotic bodies and microvesicles (Luga et al., 2012). Second, differ from other new biomarker sources, mature technologies have been developed to isolate and characterize exosomes (Lobb et al., 2015). Third, exosomes are stable in the body circulation and are easy detected in almost every potential body fluid. Last but not least, they can also mirror their underlying origin cell markers through presenting specific surface proteins (Chavez-Muñoz et al., 2008). Consequently, exosomes extracted from human body fluid are under extensive investigation to further allow for better treatment choices. For example, fifty-three localized BC patients received neoadjuvant therapy, after analyzing exosomal miRNAs and circulating tumor cells as potential biomarkers, researchers found that higher expression of exosomal miRNA-21, miRNA-222, and miRNA-155 were strongly correlated with different diagnosis and treatment response in diverse patients (Rodríguez-Martínez et al., 2019). In addition, a set of exosomal miRNAs in circulating system targeting immune maturation related pathway, predicted poor response to neoadjuvant therapy preceded to chemotherapy in TNBC (Salvador-Coloma et al., 2020). Besides, another investigation showed that the level of lncRNA-HOTAIR in circulatory exosome was positively associated with the status of HER2 in BC tissues (Wang Y. L. et al., 2019).

Not only exosomes, other extracellular vesicles are found to play a role in liquid biopsy. With clinical validation, a novel technology using nanopatterned microchips was reported to monitor tumor progression and metastasis. This technology demonstrated matrix metalloproteases-14 on extracellular vesicles to detect cell invasiveness *in vitro* (Zhang et al., 2020). However, an investigation into the comparison between extracellular DNA and circulating tumor DNA found that while copy number variants were both detectable in two kinds of DNA, circulating tumor DNA have better sensitivity in serial monitor of BC (Ruhen et al., 2020).

To sum up, different content of bioliquid exosomes may applied in diagnosing and predicting treatment response of patients with BC.

### Exosomes as Potential Carriers

Exosomes are promising natural carriers of biomolecules or anti-tumor agents because they decrease clearance in the circular system and increase specificity toward target cells after modification by surface proteins and engineered targeting methods. For example, bone marrow-derived mesenchymal stem cell-released exosomes were designed to be loaded with anti-miRNA drugs and efficiently transferred into BC cells *in vitro*. Delivery of anti-miRNA-142-3p downregulated levels of miRNA-142-3p, which further increased the transcription of certain target genes and consequently led to reduced tumorigenicity (Naseri et al., 2018). In addition, exosomes can be used as novel carriers to strengthen BC treatment efficiency after loading with specific agents and subsequent uptake by target cells. For instance, M1-polarized macrophages secrete exosomes that are used as paclitaxel carriers with a specific nanoformulation that induces a pro-inflammatory environment. After coincubation with the complex, expression of caspase-3 was increased in 4T1 BC cells in mice, which indicated the role of exosomes in enhancing the anti-tumor effects of chemotherapeutics (Wang P. et al., 2019). One of the advantages of exosomes is their low toxicity; doxorubicin-loaded exosomes were less toxic than pure doxorubicin but did not exhibit decreased drug efficacy as demonstrated both *in vitro* and *in vivo* (Toffoli et al., 2015).

Exosomes were also engineered to retarget recipient cells to increase the efficiency of delivery and improve anti-tumor ability. TNBC has a poor prognosis due to a lack of appropriate therapeutic targets, and clinical chemotherapeutics are often restricted by easy drug removal. As a result, exosomes that possess traits of delivering cargos without elimination in circular systems were designed to improve the efficacy of chemotherapeutics. In a recent study, macrophages released exosomes with coated nanoparticles engineered by a peptide that was capable of specifically targeting the mesenchymal-epithelial transition factor. As this transition factor was found to be overexpressed in TNBC cells, the cellular absorption efficiency was greatly improved and further led to enhanced inhibition of tumor growth and increased tumor apoptosis (Li et al., 2020). In addition, another study demonstrated that modifying exosomes with unique monoclonal antibodies aided in improving targeted BC immunotherapy. Mechanistically, synthetic multivalent antibody redirected exosomes were developed to both target CD3 T cells and BC-associated HER2 receptors. This dual targeting induced specific anti-tumor activity as cytotoxic T cells were activated to attack HER2-expressing BC cells (Shi et al., 2020). In previous reports, exosomes were directly engineered to target BC cells. However, exosomes can be engineered and retargeted indirectly by moderating their parental cells. Consequently, let-7a miRNA was enclosed in exosomes and delivered to EGFR-expressing xenograft BC tissues after consistent intravenous injection (Ohno et al., 2013; Table 2).

**TABLE 2:** The potential roles of exosomes in treating BC.

Roles	Donor cells	Specific drug	Recipient cells	Results	References
Exosomes as complementary tools in liquid biopsy	/	/	/	Predicting outcomes	Rodríguez-Martínez et al., 2019
				Predicting resistance	Salvador-Coloma et al., 2020
				Aiding treatment choices	Wang Y. L. et al., 2019
Exosomes as potential carriers	Bone marrow-derived mesenchymal stem cells	Anti-miRNA-142-3p	BC cells	The tumorigenicity of BC was reduced	Naseri et al., 2018
	M1-polarized macrophages	Paclitaxel	BC cells	Paclitaxel-M1-exosomes exhibited higher anti-tumor effects	Wang P. et al., 2019
	Macrophages	Doxorubicin	TNBC cells	Increased inhibition of tumor growth and induced intense tumor apoptosis	Li et al., 2020
	HEK293T cells	Anti-human CD3 and anti-human HER2 antibodies	HER2-expressing BC cells	Exhibited highly potent and specific anti-tumor activity	Shi et al., 2020
Exosome-based vaccines	Her2 specific dendritic cells	Polyclonal CD4 + T cells	HER2-positive BC cells	CTL proliferation, IFN-γ production, and rescuing CTL cytotoxic effect was induced	Li R. et al., 2018
	BC cells	MiR-155, miR-142, and let-7i	Immature dendritic cells	The immune stimulation ability was enhanced and led to maturation of dendritic cells	Taghikhani et al., 2019
Implications of exosomes in metastatic BC therapy	Nischarin-positive cells	Nischarin	BC cells	BC cells motility and tumor growth were reduced	Maziveyi et al., 2019
	BC cells	CBSA/siS100A4@Exosome	Lung pre-metastatic niche	Postoperative breast cancer metastasis was suppressed	Zhao et al., 2020
	BC cells	Antibody-tagged exosomes	Macrophages	EV-triggered metastasis in BC was disrupted	Nishida-Aoki et al., 2017
	BC cells	MiRNA-126	A546 lung cancer cells	The formulation of lung metastasis was inhibited	Nie et al., 2020
Modulation of exosomal secretion	Docetaxel-resistant BC cells	d Rhamnose β-hederin	/	The formation and release of exosomes derived from docetaxel-resistant BC cells was reduced	Chen et al., 2018
	BC cells	A selective leptin receptor antagonist-the peptide LDFI (Leu-Asp-Phe-Ile)		The release of BC cell-derived exosomes was impaired	Giordano et al., 2019

### Exosome-Based Vaccines

Owing to tumor evasion of the immune system, stimulating immune responses using an exosome-based vaccine represents a promising treatment for cancer. Li R. et al. (2018) developed a novel tumor vaccine that combined HER2-specific dendritic cell-released exosomes with polyclonal CD4 + T cells. This complex demonstrated efficacy against HER2-positive tumors in an HER2 antibody therapy-resistant mouse model (Li R. et al., 2018). Therefore, this novel vaccine will likely aid in treating HER2-positive BC patients. In addition, as tumor-derived exosomes contain various components, including tumor-specific and associated antigens, it is reasonable to propose the hypothesis that tumor-derived exosomes can be used as sources to stimulate immunity against tumor cells. It was demonstrated that after exosomes were modified with miR-155, miR-142, and let-7i, the immune stimulatory ability was enhanced, leading to the maturation of dendritic cells (Taghikhani et al., 2019; Table 2).

### Implications of Exosomes in Metastatic Breast Cancer Therapy

Exosomes are thought to involved in multiple stages during invasive processes and likely contributed to early steps participated in metastasis. For example, it was collaborated that ASPH network regulated designated exosomes to enable deliver of pro-oncogenic secretome and disseminating for long-distance matastasis (Lin et al., 2019). Besides, exosome-mediated deliever of tumor-secreted miR-105 was revealed selectively destroyed tight junctions and the integrity of natural barriers which led to enhanced metastasis in BC (Zhou et al., 2014). At the same time, abundant studies also revealed exosomes exerted influence on reducing metastasis activity in BC. Sulfisoxazole is an oral antibiotic that functions as an anti-tumor and antimetastatic drug in BC xenografts. Further study using pharmacological and genetic methods and a direct binding assay demonstrated that sulfisoxazole interfered with endothelin receptor A and decreased the release of exosomes, which ultimately inhibited progression and metastasis in BC cells (Im et al., 2019). Additionally, nischarin, a tumor suppressor, was previously found to regulate early metastatic events in BC (Maziveyi et al., 2018). In further research, Maziveyi et al. (2019) revealed a novel role for nischarin in preventing BC cell motility and tumor growth by regulating Rab14 activity and consequently secreting exosomes capable of controlling tumor malignancy. In addition, a recent study suggested that antisense non-coding mitochondrial RNA was a novel target for cancer therapy in BC. After treatment with an antisense oligonucleotide, levels of antisense non-coding mitochondrial RNA were decreased in malignant BC cells. As a result, exosomes released from these BC cells were capable of reducing tumorigenic properties in recipient cells, which shed light on the positive relationship between the transfer of exosomes derived from antisense non-coding mitochondrial RNA knockdown cells and inhibition of the development of BC metastatic niches (Lobos-González et al., 2020). Similarly, researchers found that exosome-mediated siRNA presented excellent biocompatibility and high affinity toward the lung, indicating a promising strategy to inhibit metastasis in postoperative BC patients (Zhao et al., 2020). In addition, a novel therapeutic strategy exploited antibodies to target exosomes, and efficiency was confirmed both *in vivo* and *in vitro*, as antibody-tagged cancer-derived exosomes were internalized and eliminated by macrophages, leading to decreased metastatic incidence (Nishida-Aoki et al., 2017). Interestingly, specific cancer cell-released exosomes also exerted their function in treating other cancer cells. For example, exosomes released from BC cells were found in a recent study to inhibit lung cancer cell proliferation and migration as they possessed organotropic properties. Specifically, exosomes were exploited as miRNA-126 protective nanocarriers and taken up by A546 lung cancer cells, which inhibited the PTEN/PI3K/AKT signaling pathway and suppressed pulmonary tumor cell metastatic ability (Nie et al., 2020). The above findings discussed the potential role of exosomes in aiding advanced metastatic BC treatments, which give us a broaden horizon over selecting regimes (Table 2).

### Modulation of Exosomal Secretion

As discussed in previous studies, modulated exosomal secretion is also involved in improving BC clinical treatment. For example, a specific drug called d Rhamnose β-hederin was investigated to explore its effects in treating BC. It was demonstrated that this novel drug reduced the formation and release of exosomes derived from docetaxel-resistant BC cells, which contributed to reversing docetaxel resistance (Chen et al., 2018). Sulfisoxazole, an oral antibiotic, was found to be important in effectively suppressing BC metastasis and tumor cell growth by inhibiting the biogenesis and secretion of small extracellular vesicles (Im et al., 2019). Additionally, the obesity hormone leptin was found to be capable of regulating the biogenesis and generation of exosomes in both MCF-7 and MDA-MB-231 BC cells, representing a potential strategy to interrupt harmful intercommunication between cells (Giordano et al., 2019).

While exosomes offer solutions to many challenges that are encountered by clinicians in the treatment of BC, they are not devoid of obstacles. Many mechanisms inhibit their successful therapeutic application, including potential immunogenicity, the risk of stimulating metastasis, and intracellular heterogeneity (Pullan et al., 2019). With the previously published positive results, clinical trials of exosome-based delivery methods and deeper investigations are needed to confirm their clinical applicability in solid tumors (Table 2).

## Conclusion and Future Perspectives

Exosomes are a range of nanosized extracellular vesicles that play pleiotropic roles in BC development and progression. By transferring various cargos, including proteins, lipids and RNA, they are capable of modulating intercellular interplay in the tumor microenvironment. This review brings together their roles in BC therapy resistance and their mediating and communicating functions in the tumor microenvironment.

Exosomes may represent novel and accessible biomarkers of various cancers, as they are nearly ubiquitous in human cells. Moreover, there is rapidly growing evidence that exosomes are potential carriers of therapeutic agents or can be designed as vaccines to improve targeting and treatment efficiency in BC cells. Thus, the role of exosomes and their contents has become a promising research hotspot. However, which method is optimal for utilizing engineered exosomes is still unclear, and more efforts are warranted for safer and better clinical application of exosomes. Taken together, we summarize the pivotal roles of exosomes in orchestrating the tumor microenvironment and promoting BC pathological states. Fresh insights emphasizing treating BC with exosome-involved strategies are provided as well.

## Author Contributions

XW: conceptualization and writing–original draft preparation. CS: writing–review and editing. XH: visualization. JL and ZF: supervision. WL: project administration. YY: funding acquisition. All authors read and agreed to the published version of the manuscript.

## Conflict of Interest

The authors declare that the research was conducted in the absence of any commercial or financial relationships that could be construed as a potential conflict of interest.

## Publisher’s Note

All claims expressed in this article are solely those of the authors and do not necessarily represent those of their affiliated organizations, or those of the publisher, the editors and the reviewers. Any product that may be evaluated in this article, or claim that may be made by its manufacturer, is not guaranteed or endorsed by the publisher.
